# Belgian dispatchers' telephone cardiopulmonary resuscitation protocol training: an evaluation study

**DOI:** 10.1186/cc9709

**Published:** 2011-03-11

**Authors:** A Ghuysen, M El FAssi, S Stipulante, V D'orio

**Affiliations:** 1 CHU Liège - ULG, Liege, Belgium; 2 SPF Public Health Services, Liege, Belgium

## Introduction

Early bystander cardiopulmonary resuscitation (CPR) is one of the most effective interventions in improving outcome from sudden out-of-hospital cardiac arrest. However, despite large-scale community training programs, citizen-CPR rates have been persistently low. Therefore, a recent report of the 2010 European Resuscitation Council guidelines has re-emphasized the need for dispatchers to be specifically trained in starting telephone CPR protocol for suspected cardiac arrest. In accordance, 112 Belgian dispatchers have been trained for resuscitation assistance by telephone, using a specific protocol named ALERT (Algorithme Liègeois d'Encadrement à la Réanimation Téléphonique). The present work evaluates the educational aspects of this recent implementation.

## Methods

This was a prospective multicentric study including all French-speaking dispatchers in Belgium (*n *= 140). The aim was to assess the added value of the training, based on the model of Donald Kirkpatrick that allowed gathering information about perceptions of dispatchers, their satisfaction with the training and their actual ability to apply the protocol.

## Results

Dispatchers had a good pre-existing overall knowledge of CPR (80%), which was nevertheless significantly increased by the training (97%). There was a significant improvement in perceptions of dispatchers regarding their assistance skills (+44%). The training provided a significant improvement in staff perceptions on applicability of the approach on the field, and impacts for the victims. Participants (96%) were generally satisfied with the training. Finally, participants' knowledge on public health issues (33%), basic life support (+17%) and dispatching protocol (+19%) was significantly improved.

## Conclusions

French-language federal training in the 100/112 dispatching centers significantly improves dispatchers' perceptions and knowledge of assistance to resuscitation by the ALERT protocol. Such results reinforce the pivotal role of standardized protocols and training in art and science medical dispatching.
